# Long chain fatty acids alter the interactive binding of ligands to the two principal drug binding sites of human serum albumin

**DOI:** 10.1371/journal.pone.0180404

**Published:** 2017-06-29

**Authors:** Keishi Yamasaki, Saya Hyodo, Kazuaki Taguchi, Koji Nishi, Noriyuki Yamaotsu, Shuichi Hirono, Victor Tuan Giam Chuang, Hakaru Seo, Toru Maruyama, Masaki Otagiri

**Affiliations:** 1 Faculty of Pharmaceutical Sciences, Sojo University, Kumamoto, Japan; 2 DDS Research Institute, Sojo University, Kumamoto, Japan; 3 Department of Clinical Pharmacy, Yokohama University of Pharmacy, Yokohama, Japan; 4 School of Pharmacy, Kitasato University, Tokyo, Japan; 5 School of Pharmacy, Faculty of Health Sciences, Curtin University, Perth, Australia; 6 Graduate School of Pharmaceutical Sciences, Kumamoto University, Kumamoto, Japan; Islamic Azad University Mashhad Branch, ISLAMIC REPUBLIC OF IRAN

## Abstract

A wide variety of drugs bind to human serum albumin (HSA) at its two principal sites, namely site I and site II. A number of reports indicate that drug binding to these two binding sites are not completely independent, and that interactions between ligands of these two discrete sites can play a role. In this study, the effect of the binding of long-chain fatty acids on the interactive binding between dansyl-L-asparagine (DNSA; site I ligand) and ibuprofen (site II ligand) at pH6.5 was examined. Binding experiments showed that the binding of sodium oleate (Ole) to HSA induces conformational changes in the molecule, which, in turn, changes the individual binding of DNSA and ibuprofen, as well as the mode of interaction between these two ligands from a ‘competitive-like’ allosteric interaction in the case of the defatted HSA conformer to a ‘nearly independent’ binding in the case of non-defatted HSA conformer. Circular dichroism measurements indicated that ibuprofen and Ole are likely to modify the spatial orientation of DNSA at its binding site. Docking simulations suggest that the long-distance electric repulsion between DNSA and ibuprofen on defatted HSA contributes to a ‘competitive-like’ allosteric interaction, whereas extending the distance between ligands and/or increasing the flexibility or size of the DNSA binding site in fatted HSA evokes a change in the interaction mode to ‘nearly independent’ binding. The present findings provide further insights into the structural dynamics of HSA upon the binding of fatty acids, and its effects on drug binding and drug-drug interactions that occur on HSA.

## Introduction

Human serum albumin (HSA), the most abundant plasma protein in the blood, serves as a transport protein for endogenous substances such as fatty acids, hormones, toxic metabolites (e.g. bilirubin), bile acids, amino acids, and metal ions [1, 2]. HSA possesses an extraordinary binding capacity that includes numerous drugs [3]. The high affinity binding of drugs to HSA predominantly occurs at two specific regions on HSA [4, 5]. In the classical work of Sudlow et al., two drug binding sites, namely site I (also called warfarin binding site) and site II (benzodiazepine binding site) were proposed [6, 7]. Crystallographic studies have clarified that HSA contains three structurally similar α-helical domains, i.e., I-III, which can be further divided into subdomains A and B. The location of sites I and II have also been assigned to subdomains IIA and IIIA of HSA, respectively [1, 2, 8]. Sites I and II are generally considered to be two discrete, well-separated sites, because, in most cases, binding to these two sites takes place independently. However, allosteric interactions between ligands that bind to sites I and II have also been reported, examples of which include warfarin-diazepam [9], ceftriaxone—probenecid or diazepam [10], pinoxicam-diazepam [11], tenoxicam-diazepam [12], aspirin-amlodipine [13] and dansyl-L-asparagine (DNSA)-ibuprofen or diazepam [14].

HSA has dynamic properties [15], and its structure is readily influenced by a number of physiological factors, including pH, endogenous substances and post-translational modification [2, 3, 16]. It is well known that proton induced conformational changes in HSA occur in the physiological pH range 6–9, which are referred to as the neutral-to-base (N-B) transition [1, 2]. Around pH6, HSA essentially exists in the N conformation, whereas around pH 9, almost all the molecules are in the B conformation. It has been suggested that the N-B transition is followed by the destruction of salt bridges between domains I and III, thus causing albumin molecules to have an increased structural flexibility [17–19]. The N-B transition modifies, not only the affinity of a ligand to its binding site, but also the allosteric interactions between ligands that bind to different sites [14, 20]. For instance, we previously reported that the mode of allosteric interaction between DNSA (a site I ligand) and ibuprofen or diazepam (site II ligands) was shifted from nearly competitive to nearly independent when the pH was increased from 6.5 to 8.2 [14]. Such a pH dependent change in the interaction mode was considered to be due to a change in spatial relationships between the sites (i.e. subdomains or domains) as a result of the N-B transition [14].

HSA is the primary binding protein for fatty acids in the blood, acting as a carrier to transport them to and from tissues according to metabolic demands [2, 21, 22]. Under physiological conditions, circulating albumin carries approximately 0.1–2.0 moles of fatty acid per mole of protein [23]; this number increases to about 6 during fasting or maximum exercise [24, 25], or in patients with diabetes or cardiovascular disease [26, 27]. Crystallographic data revealed that there are 7 binding sites for fatty acids (labelled FA1-FA7) and that these sites are asymmetrically distributed over all the domains and the domain interfaces of the albumin molecule [28, 29]. The locations of some fatty acid binding sites overlap with site I (i.e., FA7) and site II (i.e., FA3 and FA4). Krenzel et al., using NMR spectroscopic analyses, revealed that sites I and II correspond to low-affinity binding sites for oleic acid, the most abundant fatty acid in plasma [30]. The binding of a long chain fatty acid such as oleic acid is known to cause both local structural changes and a large conformational change in HSA, involving the rotation of both domains I and III relative to domain II [8, 28, 31]. In general, the affinity of site I ligands for HSA allosterically increases by the presence of up to 3 moles of oleate (Ole) to one mole of HSA [32, 33]. A conformational change in the N-B transition has been reported to be similar to that induced by fatty acid binding to HSA [22, 34]. Indeed, The effect of Ole on the binding of site I ligands is similar to the effect caused by the N-B transition [35–37]. These findings led us to conclude that, in addition to pH, long chain fatty acids could also affect the ligand binding to sites I and II, and induce allosteric interactions between these sites through conformational change in the molecule. In the present work, the effect of the binding of long-chain fatty acids on the interactive binding between DNSA (site I ligand) and ibuprofen (site II ligand) at pH6.5 was investigated by equilibrium dialysis, circular dichroism and docking simulations.

## Materials and methods

### Materials

HSA was donated by the Chemo-Sera-Therapeutic Research Institute (Kumamoto, Japan). It was defatted with activated charcoal in an aqueous solution that had been adjusted to pH 3 with H_2_SO_4_ at 0°C, dialyzed against de-ionized water and then freeze-dried, as originally described by Chen^23^. The molecular mass of HSA was assumed to be 66,500 Da. The HSA used in this study gave only one band in SDS-PAGE. Dansyl-L-asparagine (DNSA) was purchased from Sigma Chemical Co. (St. Louis, MO, U.S.A.). Ibuprofen and sodium oleate (Ole) were obtained from Wako Chemical Co. (Osaka, Japan). All other chemicals were of analytical grade. All buffers used were prepared with sodium phosphate dibasic and sodium phosphate monobasic salts. All ligand molecules were first dissolved in methanol; the final concentration of methanol was less than 1% (v/v).

### Equilibrium dialysis

Equilibrium dialysis experiments were performed using 2 mL Sanko plastic dialysis cells (Fukuoka, Japan). The two cell compartments were separated by Visking cellulose membranes. Aliquots (1.5 mL) of samples were dialyzed at 25°C for 12 h against the same volume of buffer solution. After reaching equilibrium, the concentration of free ligands in the buffer compartment was determined by HPLC. The HPLC system consisted of a Hitachi 655A-11 pump, Hitachi 655A variable wavelength UV monitor and a Hitachi F1000 variable fluorescence monitor. The stationary phase was a LiChrosorb RP-18 column (Cica Merck, Tokyo, Japan) and was maintained at 40°C. The mobile phase consisted of 5 mM phosphate buffer (pH 7.7)-acetonitrile (77:23 v/v) for DNSA and ibuprofen assay. A fluorescence monitor was used for DNSA detection (excitation at 330 nm and emission at 550 nm). Since linearity was observed at much lower concentration of DNSA, we conclude that inner filter effects were minimal within the DNSA concentrations measured by HPLC. Ibuprofen was detected at a fixed wavelength, 220 nm using a UV monitor. The adsorption of ligands to the membrane and/or the dialysis apparatus was negligible since no adsorption was detected in equilibrium dialysis experiments in the absence of albumin. The volume shift after equilibrium dialysis was corrected according to the method of Giacomini et al. [38]

### Binding data analysis

Binding parameters were estimated by fitting the experimental data to the following equation using GraphPad PRISM^®^ Version 7 (GraphPad Software, Inc, CA, U.S.A.).

$$\text{r}=\frac{{\text{C}}_{\text{b}}}{{\text{P}}_{\text{t}}}=\frac{{\text{nKC}}_{\text{f}}}{\text{1}+{\text{KC}}_{\text{f}}}$$ (1)

where r is the number of moles of ligand bound per mole protein. P_t_ is the protein concentration, and C_b_ and C_f_ are the bound and unbound ligand concentrations, respectively. K and n are the association constant and the number of binding sites for the high-affinity binding site, respectively. All experiments and analyses were performed using the condition, r < 0.4, to minimize ligand binding to any low-affinity binding sites.

In order to simultaneously estimate the interaction mode between two ligands, A and B, which are binding to each primary binding site of HSA, the data were treated according the method of Kragh-Hansen [3, 39]. In this method, when the number of high binding sites for ligands A and B (n) is 1, the number of moles of ligands A and B bound per mole protein, r_A_ and r_B_ can be described as follows:

$${\text{r}}_{\text{A}}=\frac{{\text{A}}_{\text{b}}}{{\text{P}}_{\text{t}}}=\frac{{\text{K}}_{\text{A}}{\text{A}}_{\text{f}}+{\chi \text{K}}_{\text{A}}{\text{K}}_{\text{B}}{\text{A}}_{\text{f}}{\text{B}}_{\text{f}}}{\text{1}+{\text{K}}_{\text{A}}{\text{A}}_{\text{f}}+{\text{K}}_{\text{B}}{\text{B}}_{\text{f}}+{\chi \text{K}}_{\text{A}}{\text{K}}_{\text{B}}{\text{A}}_{\text{f}}{\text{B}}_{\text{f}}}$$ (2)

$${\text{r}}_{\text{B}}=\frac{{\text{B}}_{\text{b}}}{{\text{P}}_{\text{t}}}=\frac{{\text{K}}_{\text{B}}{\text{B}}_{\text{f}}+{\chi \text{K}}_{\text{B}}{\text{K}}_{\text{A}}{\text{A}}_{\text{f}}{\text{B}}_{\text{f}}}{\text{1}+{\text{K}}_{\text{B}}{\text{B}}_{\text{f}}+{\text{K}}_{\text{A}}{\text{A}}_{\text{f}}+{\chi \text{K}}_{\text{B}}{\text{K}}_{\text{A}}{\text{A}}_{\text{f}}{\text{B}}_{\text{f}}}$$ (3)

where A_b_ and B_b_ are the bound concentration of ligands A and B, respectively. K_A_ and K_B_ are the binding constant of ligand A and B, respectively. A_f_ and B_f_ are the free concentration of ligand A and B, respectively, and P_f_ is the concentration of free protein. χ is the coupling constant. In these equations, independent binding of the two ligands is characterized by χ = 1, while a competitive interaction results in χ = 0. χ > 1 and 0 < χ < 1 express cooperative- and anti-cooperative interaction between ligands A and B on the protein, respectively.

### CD measurements

CD measurements were made using a Jasco model J-720A spectropolarimeter (Tokyo, Japan), and a 10 mm cell at 25°C. Induced ellipticity was defined as the ellipticity of DNSA-HSA mixture minus the ellipticity of HSA alone within the same wavelength region (300nm-450nm) and is expressed in degrees. No induced ellipticities for Ole and ibuprofen were observed under the present experimental conditions. Furthermore, Ole and ibuprofen did not cause any significant change in the molar ellipticity of HSA within the wavelength region monitored.

### Docking simulation of DNSA and ibuprofen to HSA in the absence and presence of Ole

The X-ray structures of HSA were obtained from the RCSB Protein Data Bank (PDB IDs: 1gni, 2xbg, 2xvu, 2xvv, and 4g03), where 1gni is the structure of HSA complexed with seven molecules of oleic acid, 2xbg is HSA with two molecules of ibuprofen, 2xvu is HSA with two molecules of DNSA, 2xvv is HSA with two molecules of DNSA and six molecules of myristic acid and 4g03 is HSA without any ligand. Using a multi-template homology modeling technique, we constructed three models: HSA with one ibuprofen bound at site II (ibuprofen/HSA), HSA with one DNSA bound at site I and one ibuprofen bound at site II (DNSA-ibuprofen/HSA), and one DNSA bound to HSA site I and one ibuprofen bound to site II with 4 oleic acids (replacing 3 of 7 Ole with DNSA and ibuprofen) (Ole-DNSA-ibuprofen/HSA). The homology modellings were carried out using Prime 4.1 (Schrödinger, LLC., New York, NY, USA, 2015).

Because the obtained models were truncated, the N- and C-terminal residues were protected by the acetyl group (ACE) and a methylamino group (NMA), respectively. The ionization states of ligands were determined based on pKa calculations (ADMET Predictor 7.2, Simulations Plus, Inc., Lancaster, CA, USA, 2015). The ionized states of the amino acid residues and the hydrogen-bonding networks in the complexes were prepared using Protein Preparation Wizard of Maestro 10.3 (Schrödinger, LLC.). We kept the same ionization states in order to facilitate the comparison of the ligand-HSA complexes. The complexes were optimized to reduce the root mean square of the gradients of potential energy below 0.05 kJ/mol using MacroModel 10.9 (Schrödinger, LLC.). The optimizations were performed using the MM-GB/SA model to consider the hydration of the complexes. For the energy calculations, the OPLS3 force field was used without a cut-off. In order to evaluate the difference between the presence or absence of oleic acid, the interactions of ibuprofen with HSA were evaluated using the extra precision (XP) of Glide 6.8 (Schrödinger, LLC.) as the scoring function. Here, DNSA and oleic acid were treated as part of the protein. For the purpose of determining the origin of allosteric effects, the XP scores were divided into the following terms: hydrogen-bond (HBond), reward for hydrophobic enclosure (PhobEn), reward for low molecular weight (LowMW), rotatable bond penalty (RotPenal), van der Waals energy of lipophilic atoms (LipophilicEvdw), electrostatic (Electro).

## Results

### Effect of Ole on the individual binding of DNSA and ibuprofen

**Fig 1:**
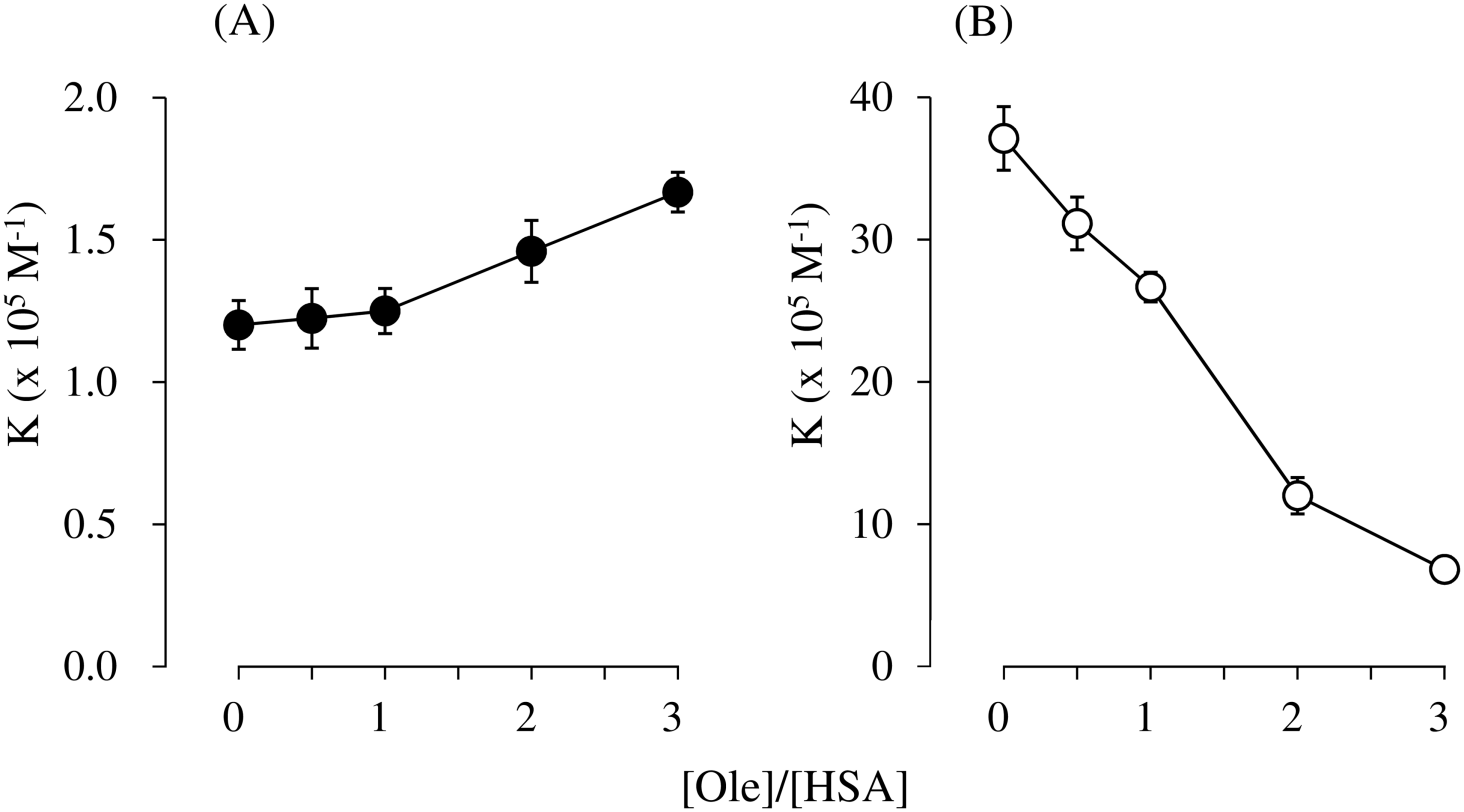
Effect of Ole on the primary association constants (K) of DNSA (A, Closed circles) and ibuprofen (B; Open circles) at pH 6.5 and 25°C. Number of primary binding sites (n) was 1 for each system. The concentration of HSA was 40μM. The results are the mean ± S.D. for at least three observations.

Fig 1 shows the primary association constants for DNSA and ibuprofen bound to HSA in the presence of increasing concentrations of Ole at pH 6.5. An increase in the Ole to HSA ratio gradually increased the binding affinity of DNSA but caused a corresponding decrease in the binding affinity of ibuprofen. In all cases, the binding of DNSA, as well as ibuprofen, took place at a single high-affinity site (n = 1).

### Effect of Ole on interaction mode between DNSA and ibuprofen bound to HSA

**Fig 2:**
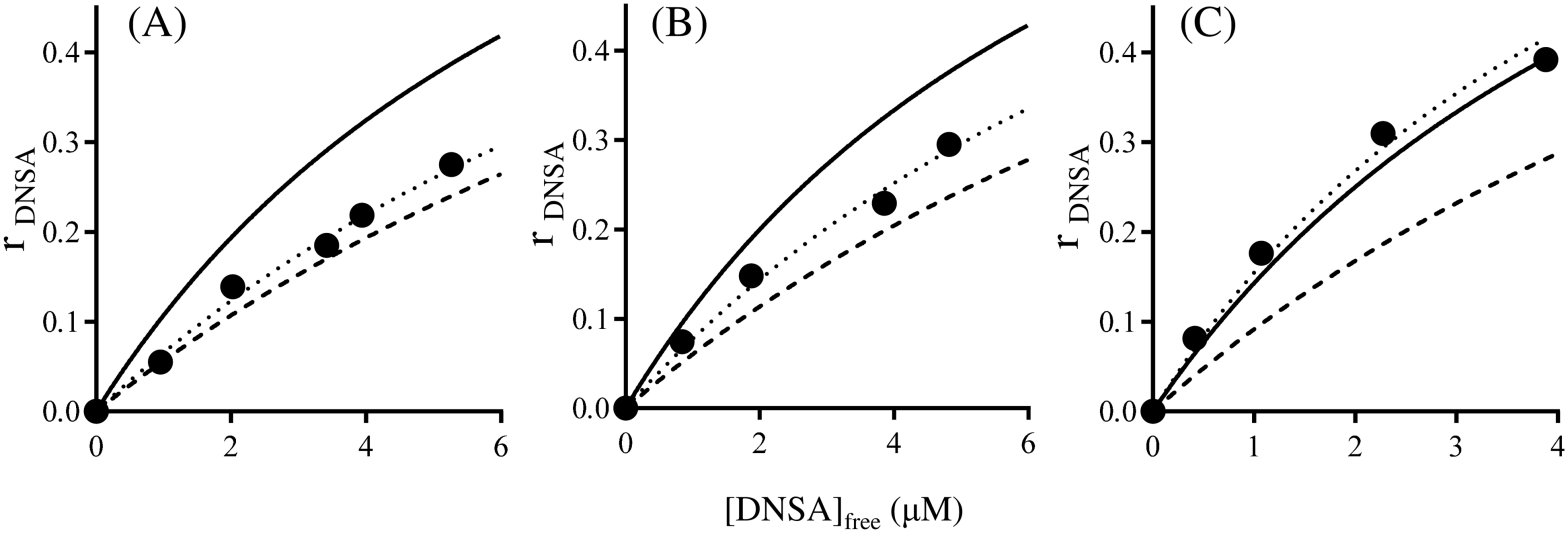
Effects of Ole on the binding of DNSA (4–24μM) to HSA (40μM) in the presence of ibuprofen at pH 6.5 and 25°C. The concentration ratio of Ole to HSA are 0 (A), 1 (B) and 3 (C). Closed circles are the experimental values for DNSA binding in the presence of ibuprofen (A, B; 20μM, C; 24μM). Solid line represents theoretical curves assuming the independent binding of the two ligands. Broken line represents theoretical curves assuming competitive binding between DNSA and ibuprofen. Dotted line represents theoretical curves assuming anti-cooperative (allosteric) interaction between DNSA and ibuprofen. All theoretical curves were constructed using the association constant for each ligand (A; DNSA 1.2×10^5^ M^-1^, ibuprofen 37.1×10^5^ M^-1^, B; DNSA 1.3×10^5^ M^-1^, ibuprofen 26.7×10^5^ M^-1^, C; DNSA 1.7×10^5^ M^-1^, ibuprofen 6.8×10^5^ M^-1^).

**Fig 3:**
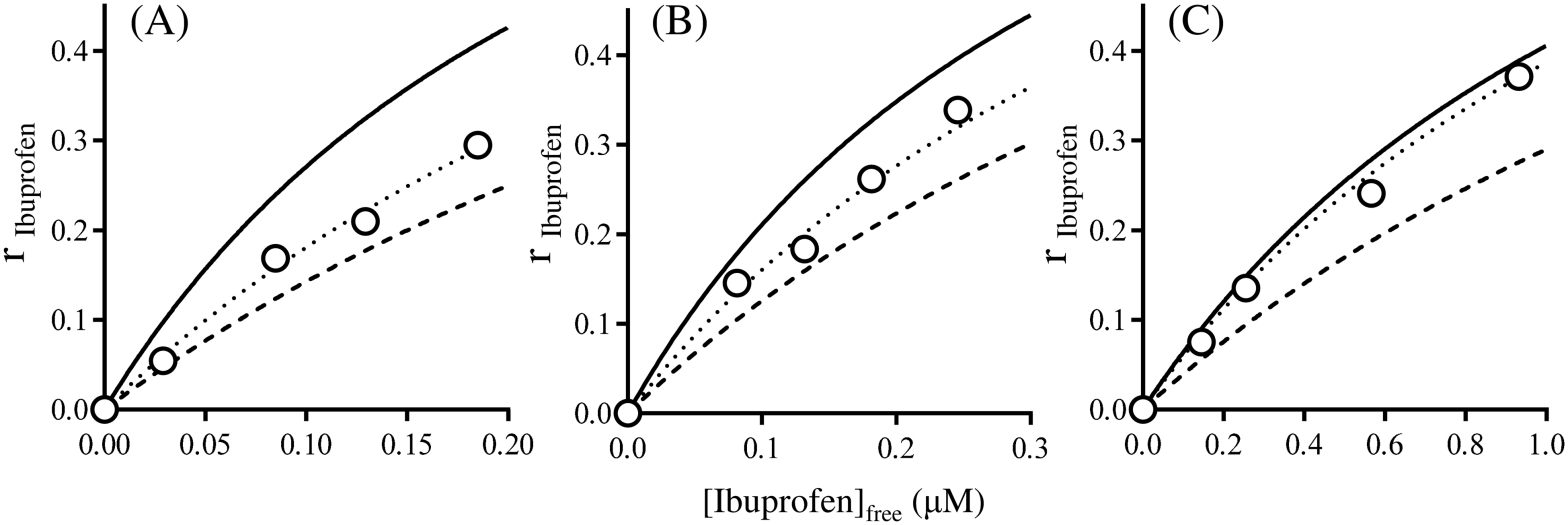
Effects of Ole on the binding of ibuprofen (4–24μM) to HSA (40μM) in the presence of DNSA at pH 6.5 and 25°C. The concentration ratio of Ole to HSA are 0 (A), 1 (B) and 3 (C). Open circles are the experimental values for ibuprofen binding in the presence of DNSA (24μM). Solid line represents theoretical curves assuming the independent binding of the two ligands. Broken line represents theoretical curves assuming competitive binding between ibuprofen and DNSA. Dotted line represents theoretical curves assuming anti-cooperative (allosteric) interaction between ibuprofen and DNSA. All theoretical curves were constructed using the association constant for each ligand (A; DNSA 1.2×10^5^ M^-1^, ibuprofen 37.1×10^5^ M^-1^, B; DNSA 1.3×10^5^ M^-1^, ibuprofen 26.7×10^5^ M^-1^, C; DNSA 1.7×10^5^ M^-1^, ibuprofen 6.8×10^5^ M^-1^).

**Fig 4:**
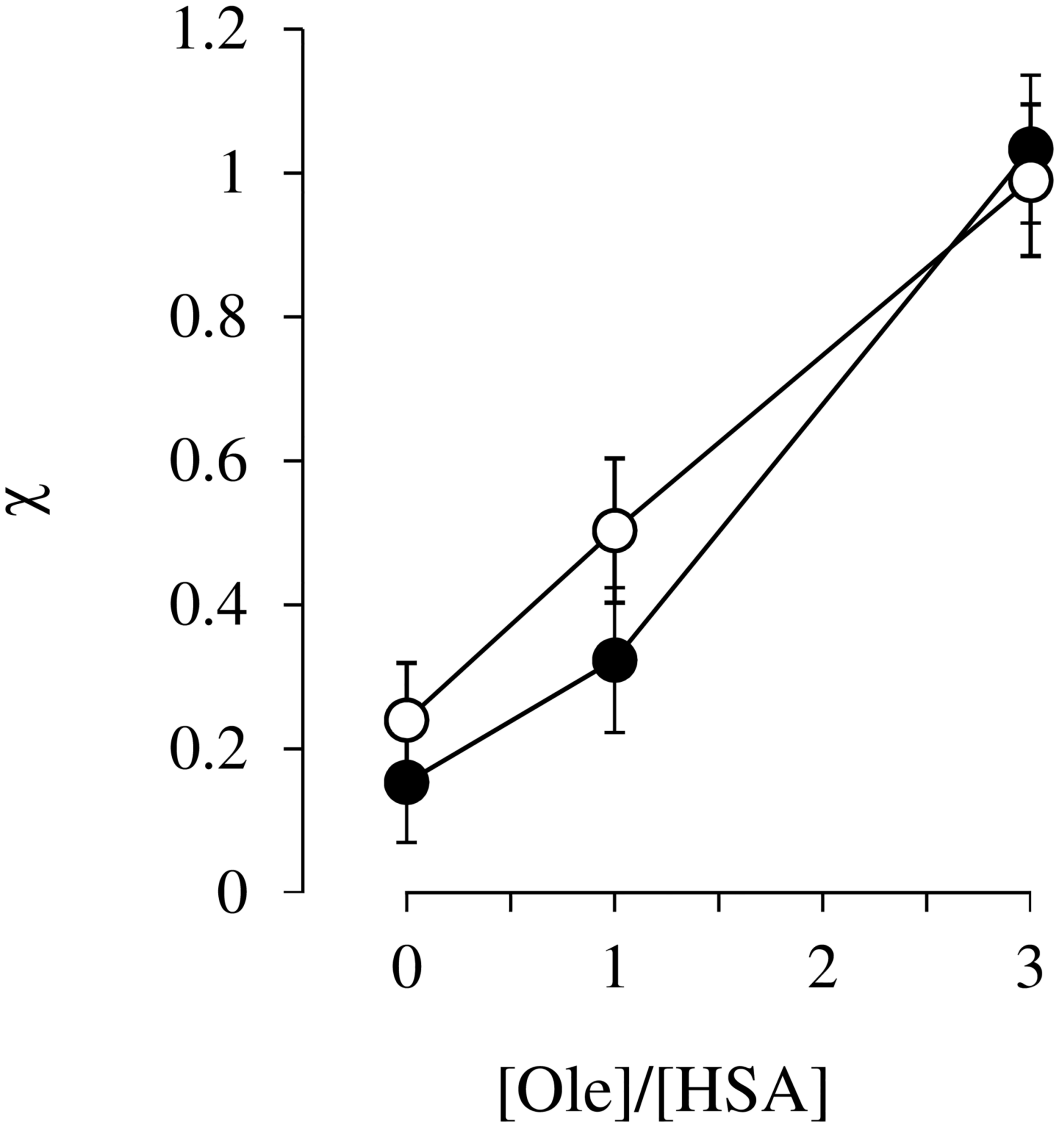
Effects of Ole on coupling constant (χ) for interactions between DNSA and ibuprofen. Closed circles are coupling constants calculated from the binding of DNSA in the presence of ibuprofen. Open circles are coupling constants calculated from the binding of ibuprofen in the presence of DNSA. The results are the mean ± S.D. for at least four determination.

The mutual binding between DNSA and ibuprofen to HSA at pH6.5 were investigated in the absence and presence of Ole. The individual association constants of these ligands to their primary binding site were used for this analysis. The interaction mode between DNSA and ibuprofen in the absence of Ole was initially investigated. As shown in Fig 2, the binding isotherm for DNSA in the presence of ibuprofen (Fig 2A) was fairly close to the theoretical curve assuming that these two ligands were competing for a common site. These findings are the same as those reported in our previous study [14]. Secondly, the effect of Ole on the interaction mode was examined. The experimental data for 1:1 and 1:3 Ole:HSA ratios were reasonably close to the curve obtained by simulation, assuming the existence of anti-cooperative and independent binding between DNSA and ibuprofen to HSA, respectively (Fig 2B and 2C). The changes in the binding isotherms of ibuprofen in the absence and presence of DNSA were similar to those for DNSA (Fig 3). As shown in Fig 4 the concentration of Ole influences the values for the coupling constants (χ) based on the data in Figs 2 and 3. The χ values for DNSA-ibuprofen interactions on HSA were increased from near the lower limit level to near the higher limit level with increasing concentrations of Ole.

### Characteristics of CD spectra

**Fig 5:**
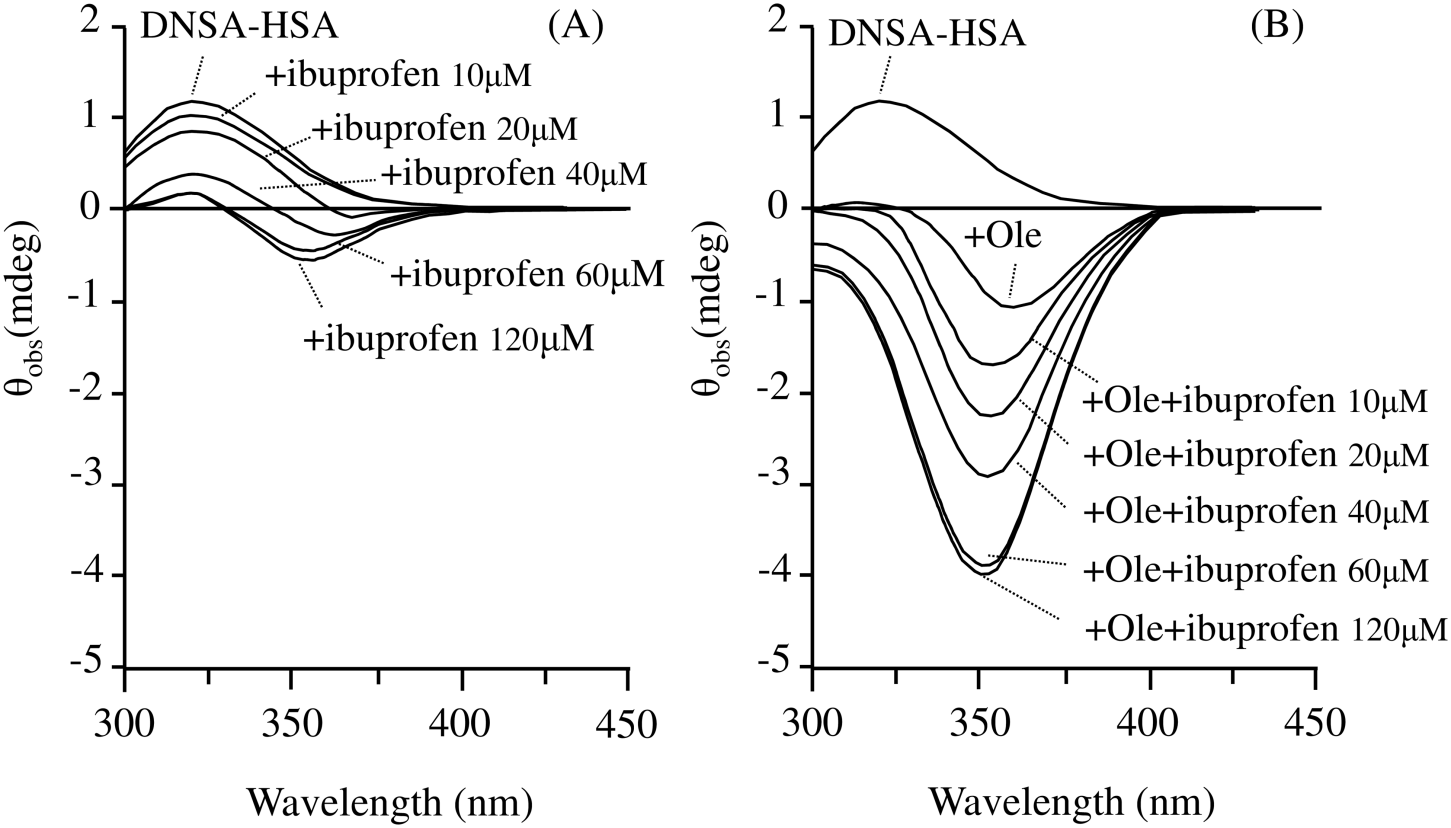
Effect of ibuprofen on CD spectra of the DNSA-HSA system in the absence (A) and presence (B) of Ole at pH6.5. Concentrations of HSA, DNSA, Ole and ibuprofen were 40μM, 40μM, 120μM, 10–120μM, respectively.

The binding of DNSA to HSA at pH 6.5 resulted in the induction of a positive Cotton effect with the maximal effect at around 325 nm (Fig 5A; DNSA-HSA). The intensity of the positive Cotton effect decreased with increasing ibuprofen concentration, and a new negative Cotton effect was generated at around 370 nm, the intensity of which increased with a shift in the maximum wavelength to 360nm (Fig 5A; + ibuprofen10~120μM). In the presence of Ole (Fig 5B; + Ole), the positive Cotton effects observed for the DNSA-HSA system disappeared, whereas a negative Cotton effect at around 360 nm was induced. The additions of ibuprofen caused a marked increase in the intensity of the negative Cotton effect observed in DNSA-HSA-Ole system with a shift in the maximum wavelength to 350 nm (Fig 5B; + ibuprofen10~120μM).

### Docking simulations of DNSA and ibuprofen to HSA in the presence of Ole

**Fig 6:**
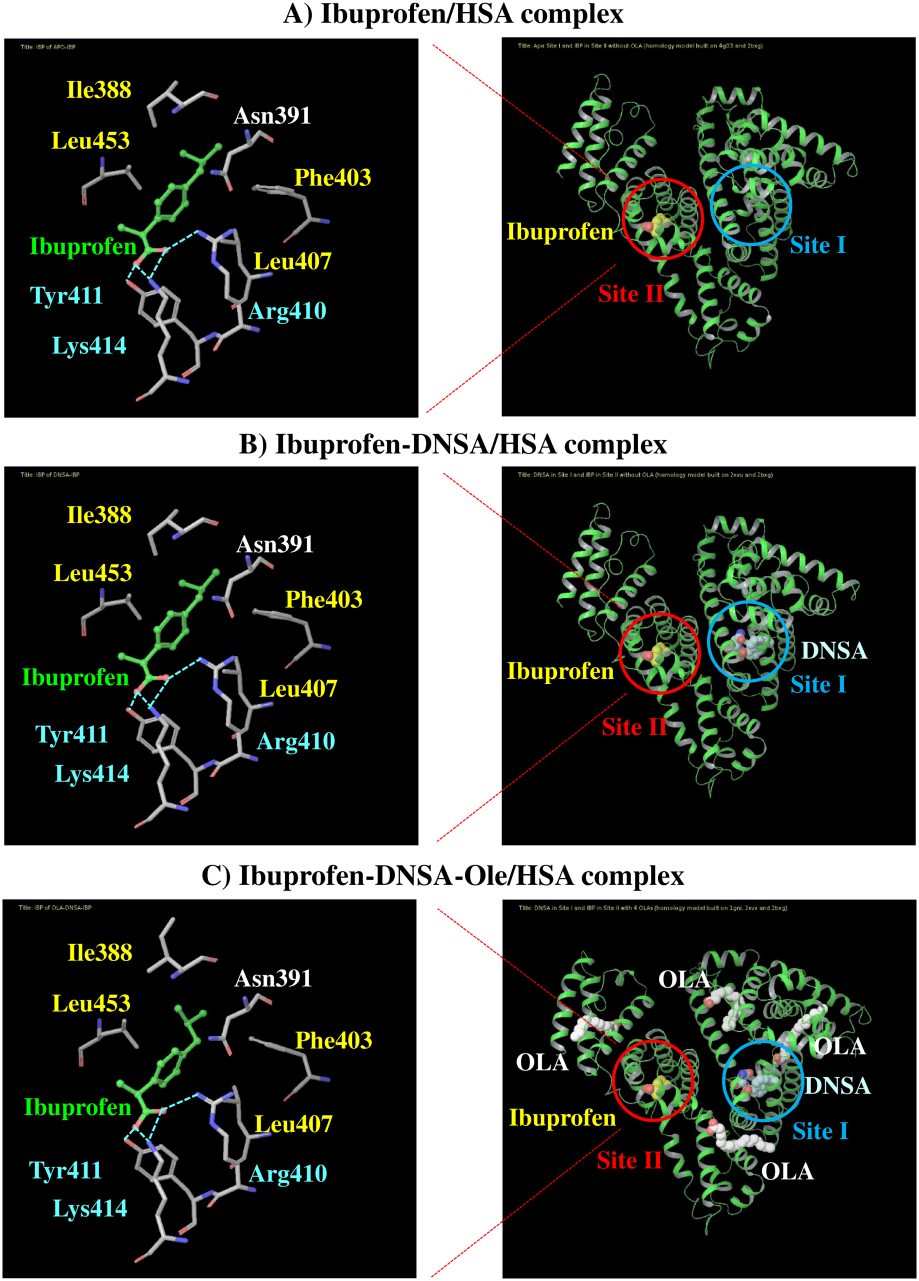
Molecule of ibuprofen at site II in the absence (A) and presence of DNSA and/or Ole (B and C). The optimized docking poses for ibuprofen are represented in green sticks. Hydrogen bonds were highlighted by bright-blue colored broken lines. Right-blue and yellow letters represent residues that interact with ibuprofen by hydrogen bonding and hydrophobic interactions, respectively.

Docking simulations of complexes of HSA, DNSA and/or ibuprofen in the absence and presence of Ole was performed. Docking poses of ibuprofen in site II indicated that hydrogen bonding (with Arg410, Tyr411, Lys414) and hydrophobic interactions (with Ile388, Phe403, Leu407, Leu453) were both involved in ibuprofen-site II interactions, even in the presence of DNSA and Ole (Fig 6). The binding affinity of ibuprofen for site II was then predicted by the XP score (Table 1). The XP score for the ibuprofen-HSA system in the absence of Ole was increased by DNSA binding, indicating a decreased affinity for ibuprofen. This increased XP score was accompanied by an increase in the score for electrostatic interactions (Table 1; Electro). Meanwhile, the XP score for the ibuprofen-DNSA-HSA system was slightly decreased by Ole binding. For the simulation of ibuprofen-HSA complex, the distance between the α carbons of Trp214 and Tyr411 which are located in sites I and II, respectively was 27.09Å. The distance in DNSA-ibuprofen-HSA and DNSA-ibuprofen- Ole -HSA complexes was elongated to 27.37 and 27.62Å, respectively.

**Table 1:** Docking data of ligands-HSA complexes.

Complexes	Docking score (kcal/mol)							Distance between α carbons of Trp214 and Tyr411 (Å)
	HBond	PhobEn	Low MW	Rot Penal	LipophilicEvdW	Electro	XP Score	
Ibuprofen-HSA	-2.3	-1.5	-0.5	0.2	-2.9	-2.6	-9.5	27.09
Ibuprofen-DNSA-HSA	-2.3	-1.1	-0.5	0.3	-2.9	-1.2	-7.6	27.37
Ibuprofen-DNSA-Ole-HSA	-2.4	-1.3	-0.5	0.3	-3.2	-1.2	-8.2	27.62

## Discussion

The crystallographic data for HSA revealed that sites I and II are located in subdomains IIA and IIIA, respectively. [1, 8] The data also clearly showed that these subdomains shared a common interface which is stabilized by hydrophobic and salt bridge interactions [1, 8]. We previously reported on the existence of an allosteric interaction between DNSA and ibuprofen or diazepam, that bind to site I and site II, respectively, at pH 6.5 [14]. This interaction mode was described as a “competitive-like” antagonism. Changing the pH from 6.5 to 8.2 modified the interaction mode from “competitive-like” to “nearly independent”, presumably due to a change in the spatial relationship between sites I and II caused by a pH dependent conformational change in HSA, namely, the N-B transition [14].

An X ray crystallographic study revealed that Ole binds to ligand binding sites FA1-7 of HSA, and that the structure of the defatted HSA undergoes a large conformational change [29]. An Ole-induced conformational change is known to increase the affinity of warfarin (a site I ligand) [32, 33], but decreases the affinity of diazepam (a site II ligand) [40]. Similarly, the present findings indicate that a gradual increase and decrease in the affinities of DNSA and ibuprofen, respectively, occur on the addition of Ole (~1:3 Ole:HSA ratio) (Fig 1). Krenzel et al. investigated the binding of Ole to HSA using 2D NMR [30]. They observed three resonances corresponding to high affinity sites at 1:1 and 1:2 Ole:HSA ratios, which were different from sites I and II. At a 1:3 Ole:HSA ratio, five additional resonances corresponding to sites I and II were evident and the resonance for site I was the most prominent. It thus appears that sites I and II function as low affinity sites for Ole at a 1:3 Ole:HSA ratio and thereby could partially prevent the binding of DNSA and ibuprofen, although an increased binding of DNSA was observed in the present study (Fig 1A). Concentration ratios of Ole to HSA, 0, 1 and 3 were used in this study, since circulating albumin carries approximately 0.1–2.0 moles of fatty acid per mole of protein [23] and this number increases to about 6 during fasting or conditions of maximum exercise [24, 25], or in patients with diabetes or cardiovascular disease [26, 27]. The experimental concentrations of Ole in present study were below the critical micelle concentration of Ole, and the formation of Ole-drug complexes has not been reported in these concentrations [31, 32, 39]. Ole changed, not only the individual binding of DNSA and ibuprofen to HSA, but also the interaction mode between these ligands on HSA at pH 6.5 (i.e. Ole/HSA = 0; ‘competitive-like’, Ole/HSA = 1; ‘anti-cooperative’, Ole/HSA = 3; ‘nearly independent’) (Figs 2, 3 and 4). Such a change in the mode of interaction between DNSA and ibuprofen could not be due to the competitive displacement of these ligands by Ole. Therefore, like the N-B transition, the Ole-induced conformational change appears to be the leading cause of the changes in ligand binding and the mode of interaction. At *in vivo* HSA concentrations (approximately 0.6mM), the free fraction of ligand should be less than 1.5% when its association constant is similar to that of DNSA or ibuprofen. In this condition, competitive-like interactions between ligands would cause about a 2-fold increase in each free fraction compared to that in the absence of another ligand.

Interactions between HSA and DNSA generated a Cotton effect at around 370 nm at pH 6.5 (Fig 5). Since unbound DNSA is not optically active, and HSA does not produce any Cotton effects at these wavelengths, the observed Cotton effects must be extrinsic in origin. The extrinsic Cotton effects are thought to be a result of the interaction of a ligand chromophore with an asymmetrical locus in the protein [41–44]. Thus, extrinsic Cotton effects reflect the characteristics of specific asymmetrical sites in a protein molecule and could provide information on the microenvironment of a binding site or ligand orientation in a site. In the absence of Ole, ibuprofen caused a decrease in the Cotton effect around 325 nm which can be attributed to a decreased binding of DNSA, and also generated a Cotton effect at around 360-370nm (Fig 5A). These results suggest that the decrease in DNSA binding is not due to competitive interactions, but allosteric interactions that are accompanied by a change in the orientation of DNSA at the binding site. In the presence of Ole, nearly independent binding was observed between ibuprofen and DNSA (Figs 2, 3 and 4), however ibuprofen caused a marked increase in the Cotton effect around 360-370nm (Fig 5B) despite the fact that the affinity of ibuprofen is decreased by Ole. (Fig 1B). These results indicate that the binding of ibuprofen in the presence of Ole significantly changes the orientation of DNSA, but does not affect the binding affinity of DNSA. Taking all the binding results in the absence and presence of Ole into account, the binding site of DNSA on the HSA conformer to which Ole binds may be structurally more flexible and would allow marked change in the orientation of DNSA to occur. Ryan et al. reported that a myristate-induced conformational change results in a minimal change in the position of the bound DNSA and in the amino acid residues (Lys199, Arg222 and Ala291) that are involved in hydrogen bonding interactions with DNSA within site I [45]. They also observed a conformational change in one flank of subdomain IIA, which involves the re-orientation or relocation of the side-chains of Tyr150, Glu153, Gln196 and Arg257. Such changes in the structural features surrounding DNSA by fatty acids are likely to be responsible for the flexibility or size of the site to allow a change in DNSA orientation.

Docking simulations of complexes of HSA, DNSA and/or ibuprofen in the absence and presence of Ole provided additional information regarding the effects of Ole on the interaction between DNSA and ibuprofen (Fig 6, Table 1). The XP score for ibuprofen-HSA interactions in the absence of Ole was increased by DNSA binding, suggesting a decreased affinity of ibuprofen. Indeed, the decreased binding of ibuprofen by competitive-like interactions with DNSA was also indicated in results obtained from binding experiments (Figs 2A and 3A). As observed in the docking pose, ibuprofen interacts with Arg410, Tyr411, Lys414 through hydrogen bonding, and with Ile388, Phe403, Leu407, Leu453 to form hydrophobic interactions, and these interactions were not affected by the binding of DNSA and Ole. Meanwhile, since an increase in XP score is clearly dependent on a change in electrostatic score, the allosteric displacement of ibuprofen by DNSA can be attributed to long-distance electric interference in the binding of ibuprofen to site II. Both ibuprofen and DNSA are negatively charged at pH6.5, and thereby may electrically repel each other, even though the ligands bind to different sites. The XP score for the ibuprofen-DNSA-HSA system negligibly affected by Ole binding. However, the binding data indicated that Ole not only decreases the binding of ibuprofen (Fig 1B), but also changes the mode of ibuprofen-DNSA interaction from ‘competitive-like’ antagonism to ‘nearly independent’ binding (Figs 2, 3 and 4). Therefore, a higher XP score for the ibuprofen-DNSA-Ole-HSA system compared to the ibuprofen-HSA system may reflect a decrease in the affinity of ibuprofen by Ole, but not by DNSA. The distance between the α carbons of Trp214 and Tyr411, which are amino acid residues around site I and site II, in the ibuprofen-HSA complex, 27.09Å was extended to 27.37Å in the presence of DNSA and to 27.62Å in the presence of DNSA and Ole (Table 1). Such an extension in the distance between their binding sites was induced by electric repulsions between ibuprofen and DNSA. The HSA conformation at pH6.5 (N-conformer), however, may not possess sufficient structural flexibility to permit this repulsive force to be eliminated, and would result in a decreased ligand binding. Meanwhile, an Ole-induced conformational change may cause the distance to be extended further, thus leading to the elimination of allosteric interactions of ibuprofen and DNSA as indicated in our previous study on the N-B transition, or confer more flexibility or size which can abolish repulsive forces as suggested in the CD data.

Such allosteric interactions for drugs binding to both sites I and II of HSA also occurred for combinations of warfarin-diazepam [9], ceftriaxone—probenecid or diazepam [10], pinoxicam-diazepam [11], tenoxicam-diazepam [12] and aspirin-amlodipine [13]. Not only such interaction between different ligands but the interaction between ligands binding to its high and low affinity sites has also been reported [46]. Several recent reports have suggested that ferric heme binds to the FA1 site and allosterically interacts with drugs at the FA7 site (site I) or FA6 site (a secondary ibuprofen binding site) [47–49], thereby leading to toxic concentrations of ferric heme and drugs. A change in physiological conditions such as blood pH and fatty acid concentration could affect, not only the individual binding of ligands, but also the mode of binding interaction. The physiological implication of pH and fatty acid induced conformational changes in HSA were proposed by Fanali et al. to a play role in receptor-mediated endocytosis in the delivery of fatty acids to hepatocytes [34]. They also suggested that the non-defatted HSA conformer is recognized by a putative HSA receptor and the decreased pH in endocytotic vesicles shifts the conformational equilibrium towards the defatted HSA conformer. As observed in the present study, since the allosteric interaction between sites I and II depends on the conformation of the HSA molecule (e.g. N, B, defatted and fatted conformers), changes in HSA conformers may also function in the delivery of ligands that simultaneously bind to sites I and II. The interaction between the ligands and the modification of ligand binding by a structural change in a protein have been reported in cases of several proteins [50–52]. Therefore, the findings reported here may also be useful for understanding structure-function relationships for other proteins.

## Conclusions

In this study, we attempted to clarify the effect of a long-chain fatty acid, Ole, on the allosteric interaction between DNSA and ibuprofen, which bind to two different sites, site I and site II of HSA at pH6.5, respectively. Our results indicated that an Ole-induced change in the conformation of has, not only modifies individual binding of DNSA and ibuprofen, but also modifies the mode of interaction between the ligands such that the ‘competitive-like’ allosteric interaction on the defatted HSA conformer can be changed to the ‘nearly independent’ binding on non-defatted HSA conformer. The findings suggest that long-distance electric repulsions between ligands on the defatted HSA conformer contribute to a ‘competitive-like’ allosteric interaction. Meanwhile, extending the distance between ligands and/or increasing the flexibility or size of the DNSA binding site on fatted HSA conformer could result in a change in the interaction mode to ‘nearly independent’ binding. The present findings provide further insights into the structural dynamics of HSA upon the binding of fatty acids, and its effects on drug binding and drug-drug interactions on HSA.
